# Positive and Negative Impacts of Gamification on the Fitness Industry

**DOI:** 10.3390/ejihpe13080103

**Published:** 2023-08-02

**Authors:** Fezile Ozdamli, Fulbert Milrich

**Affiliations:** 1 Department of Management Information Systems, Near East University, Nicosia 99138, Turkey; 2 Computer Information Systems Research and Technology Centre, Near East University, Nicosia 99138, Turkey; 20225777@std.neu.edu.tr; 3 Department of Computer Information Systems, Near East University, Nicosia 99138, Turkey

**Keywords:** gamification, gamified, fitness, healthiness, gym, game mechanics

## Abstract

Gamification features to motivate individuals to exercise have become a trend in the fitness sector that is gaining popularity. It is based on the idea that adding fun and competitive components to workout routines will inspire people to achieve their fitness objectives and maintain a healthy lifestyle. This research study attempts to analyze the literature that explores this concept of gamification in detail, and create a picture of how its implementation has changed fitness and healthy habits. This research incorporated the Preferred Reporting Items for Systematic Reviews and Meta-Analyses (PRISMA) approach as its research methodology. Search strategy used a set of inclusion-exclusion criteria that helped us examine through hundreds of articles identified in the Web of Science and SCOPUS databases. After exclusive and inclusion criteria, 48 articles were selected to be reviewed in detail. Results have indicated that gamification strategy is a supporting factor to overcome the difficulties of executing exercises. Also, to improve the willingness towards fitness regimens.

## 1. Introduction

Lack of exercise contributes to more than 5 million attributable deaths per year worldwide and is a risk factor for chronic disease incidence and mortality [1,2]. The World Health Organization (WHO) has declared that more than 25% of adults worldwide are physically inactive. This rate is higher in other regions around the world [3].

According to the U.S. Department of Health and Human Services published guidelines, physical exercise is vital and fundamental for individuals to have a healthier life. The increased sedentary behaviors are strongly linked to an increased risk of heart disease, high blood pressure, and all causes of mortality. The report also notes that all forms of physical activity, particularly moderate to vigorous activity, can reduce these health risks [4].

Studies show that most adults do not reach the recommended physical activity levels. Behavioral economics has been used to design scalable interventions that increase physical activity over short periods of time, but the long-term effectiveness of these strategies is uncertain [5].

These statistics highlight the need for all nations to take national measures in order to create an environment that encourages physical activity. Also, it provides an opportunity for all age groups to be physically active. The new Global Action Plan on physical activity aims to reduce 10% of inactivity by 2024 and 15% of inactivity by 2030 [3].

Physical activity is crucial for each citizen to improve the status of health physically and mentally. Wearable devices, such as smartphones and smartwatches, are able to monitor the health status and motivate individuals to conduct continuous excises. Studies have revealed that despite being aware of the health advantages of using fitness trackers to encourage themselves to stay active, many eventually stop using them [6,7].

In order to ensure the continuity of individuals’ staying active, making them fun will keep their motivation high. For this reason, gamification applications are needed. Integrating gamification features into applications on mobile or wearable devices makes physical activities fun and arouses the desire to participate and be active in communities with its challenges. Typically, this is conducted by incorporating game aspects and ideas from games into other contexts. Using game mechanics significantly boosts user engagement and increases productivity [8].

Furthermore, gamification has introduced a further level of fun in the fitness culture that was not present before. Game elements and mechanics such as scores, leaderboards, and challenges create a sense of adventure as the user/subject is always engaged in a game-like activity. By creating a sense of accomplishment, people strive to work even more to beat their high scores. Leaderboards create a sense of competition that further motivates [9]. Competing with friends is more fun and engaging [10].

It is therefore important to understand Gamified Fitness tracking apps because of their significant potential to improve public health. Fitness culture is a contemporary social phenomenon involving people who exercise and maintain physical fitness, and it has gained popularity and grown to be a significant tool for connecting with others. According to a global survey, 61% of regularly exercise individuals currently engage in “gym-type” activities [11]. 

Fitness applications collect user data and can serve many purposes, including enabling users to set fitness objectives, monitor calorie intake, obtain workout inspiration, and post updates on social media to promote healthy behavior changes. With the growth of “gym culture,” fitness app development has also developed tremendously [12]. As of 2015, fitness and health-related apps on App Store (Apple O.S.) and Google Play Store (Android) had reached more than 165,000 [13].

The apps incorporate gaming elements such as challenges, rewards, and leaderboards, increasing user satisfaction and improving adherence to physical activity [14]. Users can also track their progress over time, providing a sense of satisfaction and encouraging continued participation in physical activity. For example, Health Apps on Apple devices can track user B.M.I. Indexes the number of steps a user has taken in a day and subsequent calories burnt [13]. The Apple Watch can monitor a user’s heart rate and utilize that information to determine their mood or emotional state. The user may establish a regimen and strive to meet specified daily steps to enhance cardiovascular health. If they do not observe any progress, they may seek expert assistance from a fitness coach or a doctor. Also, in the literature review conducted, researchers stated that gamification mechanics encourage users to be more competitive in challenges related to a fitness problem [15].

Gamification has had a significant impact on the functioning of the fitness industry today. According to Mustafa et al. (2023), although mobile health applications are designed to support health behaviors and encourage physical activity, more research is needed to maintain user engagement. The researchers state that gamification elements positively ensure the continuity of active participation [16]. For these reasons, detailed research on the subject is needed. A systematic literature review includes different purposes for using gamification applications in the health field. In a systematic literature review by Arora et al., they aimed to summarize the responsibilities of the designers of gamified health practices and various ethical issues [17]. The systematic review of Neupane et al. aimed to examine the pedometer applications systematically [7]. In the literature, no systematic study has been found on studies examining the positive and negative effects of using gamification applications in the fitness field. Revealing the overall picture of its positive-negative effects and challenges can help researchers and developers decide which topics to focus more on. For this reason, it is aimed to answer the following research questions in order to determine the effectiveness of gamification and the missing gap in the literature by systematically reviewing the articles published on this subject:

- What are the positive and negative effects of using gamification applications for fitness?
- What are the challenges of using gamification applications for fitness?
- What are the recommendations for fitness gamification applications proposed by the literature?

## 2. Materials and Methods

This research conducts a systematic literature review to reflect the impacts of gamification on the fitness industry. The authors will use PRISMA to shed light on the advantages and disadvantages of gamification. The PRISMA process will immediately be followed by a systematic literature review [18].

### 2.1. Search Strategy

The sources that were evaluated and categorized according to the proposed classification context to establish the main elements of the systematic literature review. A number of keyword combinations were used by researchers. The Boolean operator “AND” was used to search for various things from the Topic search categories and uncover related studies. Similar phrases are connected using the “OR” operator to maximize coverage. Logical search strategy formula ((“gamification” OR “gamified” OR “game mechanic”) AND (“fitness” OR “gym”)). 

Some previously published articles were reviewed to learn more about the subject. Particularly, two well-known scientific databases, Web of Science and SCOPUS, were examined for this review’s systematic search in April 2023.

### 2.2. Inclusion and Exclusion Criteria

Including and exclusion criteria guarantee that the searched articles are pertinent to the research goal. In this research, the applied criterion are listed in Table 1.

The selection procedure for articles started with (381) articles. A total of 166 results were discovered in the first Web of Science database search, while 215 results were discovered in the Scopus database.

A total of 54 duplicate articles were then removed from the screening process since they were available in both databases.

After that, the abstracts (*n* = 82) were screened to apply inclusion criteria. Thirty-one articles were eliminated since they needed to be more appropriate to the topic. After reviewing full papers, three publications were eliminated since they were not directly connected to the subject. The abstracts of several studies were provided in English. Therefore, articles that had English-only abstracts, full-text access restrictions, and non-accessible articles were not included.

**Figure 1:**
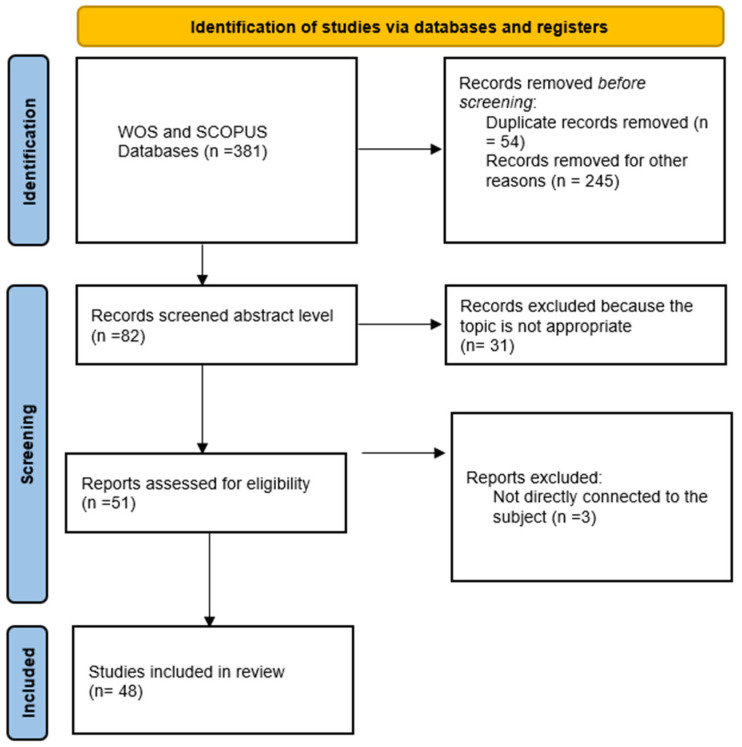
Article Selection Process with PRISMA [20].

Its researchers independently reviewed studies identified with PRISMA (Figure 1) and extracted data from included studies for prespecified variables [19]. Impacts were divided into three categories: positive, negative, and challenges. In addition to the effects, recommendations have been identified.

### 2.3. Descriptive Analysis of Articles

**Figure 2:**
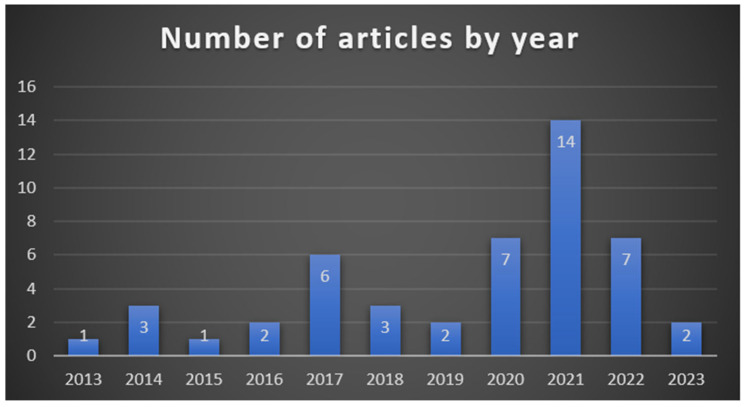
Number of Articles by Year.

The articles reviewed were collected from articles from 2013 to 2023. As shown in Figure 2 below, most articles reviewed were from 2021 (14) and the least in 2015 and 2013 (1).

As seen in the figure, the increase in research on using gamification applications in the fitness field started in 2017. With the pandemic, the number of studies has increased, and most studies on the subject were carried out in 2021.

The articles were gathered from 15 different countries and varied regions of the world, with the majority coming from the United States of America (11). In addition, it is seen that the subject is given importance in Switzerland, Spain, and Brazil. 

**Figure 3:**
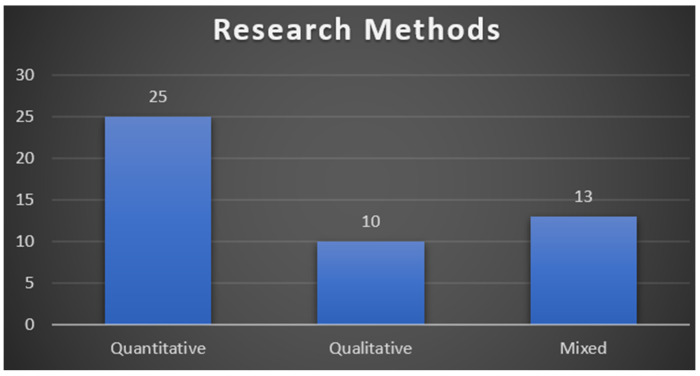
Research Method Distribution.

Previous researchers have investigated the effects of gamification in the fitness field using quantitative, qualitative, and mixed study methodologies, as shown in Figure 3. The majority of the investigations employed quantitative techniques. In addition, surveys and interviews were utilized most frequently in primary studies to understand the effects of online learning during the epidemic. Researchers combined mixed research using survey and interview approaches.

## 3. Results

This study aimed to investigate how gamification impacts the fitness industry and healthiness. As the title suggests, the research aims to shed light on the gamified fitness industry’s positive and negative effects. In addition, the authors intend to expand on the various challenges that inhibit the application of gamification. 

**Figure 4:**
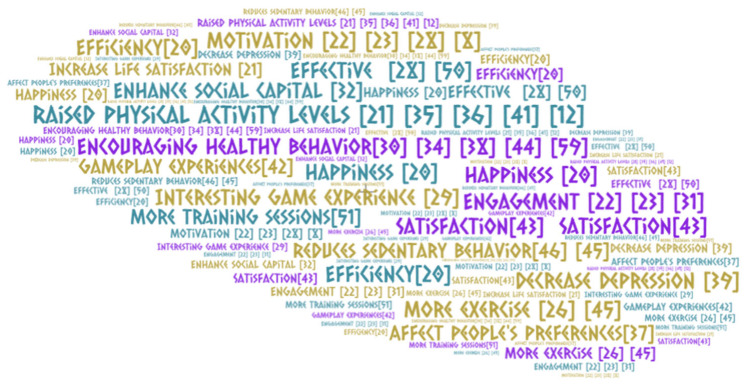
Effects of Gamification according to results.

Figure 4 summarizes the critical results of the studies included in the research. 

### 3.1. Positive Effects of Gamification

Table 2 lists the advantages of gamification applications for fitness and health, as reported in the research.

The findings from the analysis demonstrate that gamification can positively impact some aspects of physical activity, motivation [21], engagement, social interaction [23], health activities, user experiences, and the effectiveness of rehabilitation techniques. Some of these effects are increased happiness and satisfaction [22], promotion of physical activity and social connection, decreased sedentary behavior [26], enhanced attention and memory [27], and the development of practical knowledge and skills [14]. It was a common finding in the literature that applying gamification concepts in fitness motivated and increased physical activity, leading to better fitness. Exergaming is more effective among young adults [26], and although it is being applied to senior citizens as well [31], the results of this application are controversial, and the studies show conflicting results.

### 3.2. Negative Effects of Gamification

According to the literature, Table 3 shows the negative effects of gamification applications for fitness and health.

Examining the negative consequences of gamification on its use in the fitness industry reveals that, despite its beneficial effects on motivation and fitness, gamification can also have drawbacks that should be considered. Some users may find the competitive nature of gamification items motivating or stressful [36], and participants may not perceive gamified activities as real exercises [34]. Berg et al. (2020) found that relying too heavily on external rewards may reduce intrinsic motivation [35], and that focusing on short-term goals may prevent long-term behavioral changes [36]. Furthermore, the competitive nature of gamification may be detrimental to mental health [14]. Considering these potential drawbacks when developing gamification tactics that can encourage long-term involvement, intrinsic drive, and general well-being is crucial.

### 3.3. Challenges of Gamification Usage in the Fitness

The outcomes of using gamification applications in the fitness field demonstrate the many obstacles and problems connected with gamification interventions in this context. Table 4 shows the challenges of gamification applications for fitness and health, according to the literature.

Recruitment and compliance [28], accessibility to technology and digital literacy [30], user responsiveness in particular contexts [22], ethical concerns [17], and the complex relationship between behavior change techniques and actual behavior change [37], I.T. identity mediating meaningful interactions [38], designing strategies that address different user preferences [39], physical limitations [36], aesthetics improvement [40], effective gamification implementation [41], and consideration of usability concerns for particular user groups [42] are just a few of the issues that need to be taken into account. For gamification interventions in fitness to be implemented and used effectively, several issues must be resolved. 

### 3.4. Recommendations for Fitness Gamification Applications Proposed by the Literature

The studies note that gamified concepts could be better, and much more studying and research must be carried out. However, the studies provide insight and recommendations to improve user experience and results when using gamification to improve health.

- Understanding user motivations and social needs: Understanding users’ primary psychological motivators and social needs in designing gamified fitness apps [43].
- Data security: Improve user data security, as fitness apps typically capture user data. When third-party corporations or organizations track and store performance data, there is a danger that this data will be exploited or hacked somehow [14]. Therefore, there is a need to improve safety from data theft significantly.
- Usability improvements: Fixing technical issues and bugs to improve the usability of gamified fitness apps and make them more engaging [8].
- Gender inclusivity: Well-structured design of gamified apps in a way that appeals to both male and female users, especially in self-monitoring drivers [39].
- Personalization: Apps that deliver messages tailored to each individual to suggest realistic and individualized goals [44].
- User-centric analysis: Qualitative and quantitative analysis should identify how gamified interventions affect different user demographics in a user-centered design approach [45], especially the most vulnerable or at risk of negative effects [36], e.g., older adults, before implementation.
- Improved accessibility: Providing better access to clear and concise information and simplifying interaction with the app, especially for older adults, benefitting [45].

In conclusion, the investigations admit the flaws of gamified concepts and emphasize the demand for additional study and advancements. The results from this research offer suggestions for gamified fitness apps to improve user experience, data security, usability, inclusivity, personalization, user-centric analysis, and accessibility, increasing their efficacy in promoting health.

## 4. Discussion

In other systematic reviews in the literature, the duties and responsibilities of the designers of gamification applications developed for use in the field of health, which gamification elements are preferred and similar issues have been investigated. In this study, the positive and negative effects of gamification applications on the use of fitness and the suggestions of other researchers on the subject were summarized [7,8].

Gamification is a relatively new concept, so facts and information about its implementation could be clearer. However, based on the results collected from the 48 articles of the literature that was reviewed, it is clear that gamified designs are rapidly changing how to think about fitness and health.

The results obtained from the literature review support the claim that gamification improves the fitness behaviors of users. The research showed that the positive results were quite plentiful, and gamification’s positive effects outweigh the negative effects. The major benefit of gamified interventions was that it was an effective tool for motivating and promoting healthy behaviors, whether it is for children [24], older adults [45], students [26] and even sports fans [39]. It has become an effective way for people of all demographics to become healthier and more productive. These opportunities range from helping in the cardiorespiratory fitness of college students [32] to helping combat depression as a form of therapeutic intervention [40]. Also, research showed that gamification could increase motivation and adherence to exercise and overall fitness regimes and help individuals reach their health and fitness goals. Gamification elements, such as scores and leaderboards, motivate users, develop a sense of accomplishment and push them to do more, leading to a healthier and much more productive lifestyle. A rise in happiness and contentment, encouragement of physical exercise and social relationships, a reduction in sedentary behavior, increased attention and memory, and the acquisition of practical knowledge and skills are just a few of the specific results. The concept of gamification applications in fitness has been widely acknowledged in the literature as an effective way to motivate people and enhance physical activity, which has resulted in improved fitness.

Despite all its clear positive impact on the industry, it does have some isolated negative ramifications. The major challenge facing gamified interventions is getting individuals to comply [44]. It does not matter how good its impact could be if the targeted users are unwilling to partake in them. As such, educating and spreading information about gamification remains a key area that needs to be dealt with [45]. Even the apps themselves have to be designed to account for whether the users adhere to them [7]. Concerns about user responsiveness in particular contexts, ethics, the complex relationship between behavior change techniques and actual behavior change, the role of I.T. identity in facilitating meaningful interactions, designing strategies that cater to different user preferences, accommodating physical limitations, improving aesthetics, and ensuring effective gamification are among these challenges. For gamification interventions in fitness to be implemented and used successfully, several problems must be fixed. It is not easy to come to a conclusive and factual decision on a new topic that has yet to be completely studied. Further research needs to be conducted on gamified interventions, and that is a common recommendation for nearly all the articles reviewed. 

## 5. Conclusions

As a conclusion, gamification practices support the process of creating a healthier and more active society. However, more studies are needed to be carried out to find out the factors affecting gamification. The existing literature shows that the use of gamification applications positively affects behaviors regarding being healthy. For this reason, in future research and practices, cooperation with stakeholders in the fitness field should be guaranteed, other aspects of gamification for a healthy lifestyle should be determined, and studies should be continued for its greater use. To encourage our society to adapt more to healthy living activities, awareness should be raised about the accepted methodologies in the field of gamification.

In conclusion, gamification can enhance fitness by making exercise more fun, rewarding, and social. Users can have a more enjoyable experience and become inspired to exercise more frequently by adding gaming aspects to fitness apps. However, more than technology is needed to motivate individuals to exercise regularly. The success of gamification in fitness apps depends on the commensurability of game elements [46].

This article provides a general view of the studies using gamification in fitness applications in the literature. Conducting a review of the research results of 48 gamified fitness trackers, we list their positive-negative effects and the researchers’ recommendations on the subject. It is thought that our findings will help future researchers further analyze the existing gamified fitness tracker applications with the recommendations summarized from the studies and enable them to perform different applications apart from the standard gamification applications. It is also thought that it will help them to produce solutions for the determined negative effects.

Like all studies, this one has some limitations. The fact that this analysis only used two scientific databases is its most significant restriction. Databases containing research in the medical area, like PubMed, will be used in upcoming studies. The suggestions made by other researchers will also be assigned a research objective.

## Figures and Tables

**Table 1:** Inclusion and Exclusion Criteria.

Inclusion Criteria	Exclusion Criteria
✓ Articles written English	x Full text is not available online.
✓ Articles published between 2013–2023.	x Any duplicated research articles. x Articles whose main topic is only Gamification or fitness.
✓ Articles available on the WOS and Scopus.	x Articles available on the procedia. x Articles focused on other forms of Gamification
Articles in open access journals. ✓ Articles with Gamification and fitness topic.	
✓ Reviews.	

**Table 2:** Positive effects of gamification on fitness and healthiness.

Positive Effects of Gamification on Fitness and Healthiness
Incorporating Gamification into physical exercise positively affects efficiency, effectiveness, and participant satisfaction [21]. Exposure to gameful design elements increases enjoyment, satisfaction, and motivation [22]. It promotes physical activities and social interaction and combats loneliness among older adults [23]. Competitive and cooperative approaches to Gamification generate appreciation and sociality [24]. It increases training intensity and experience in exergame-based functional high-intensity interval training [25]. It reduces sedentary behaviors among students [26]. People use more health apps because they have longer attention spans and are more engaged [27]. It develops practical knowledge of users as gamification elements help them learn to develop patterns [14]. Gamification can promote physical activity and improve health [28,29]. By addressing some of the typical issues with conventional rehabilitation techniques, Gamification has the potential to be a useful tool in musculoskeletal rehabilitation [30]. It can effectively promote intrinsic motivation and engagement in older adults [31]. Gamification can make physical activity more exciting and enjoyable for employees and create a healthier workforce [24]. Gamified elements can effectively motivate users to increase their fitness and productivity within organizations [9]. A gamification-based strategy may be useful for encouraging physical activity and raising C.R.F. among college students [32]. It can counteract the often-decreasing long-term motivation of health-app users [8]. Gamified fitness apps can predict user preference according to user data [33]

**Table 3:** Negative effects of gamification on fitness and healthiness.

Negative Effects of Gamification on Fitness and Healthiness
Participants may view exergaming as entertainment rather than exercise, thus reducing their activeness [34]. It overreliance on extrinsic motivators [35]. Some users may find the competitive nature of elements demotivating or stressful [36]. It may lead to a focus on short-term goals rather than long-term behavior change [36]. It may lead to an over-emphasis on competition, adversely affecting mental health [14].

**Table 4:** Challenges of Using Gamification.

Challenges of Using Gamification
Recruiting participants and ensuring compliance with the gamification intervention [28]. Access to technology and digital literacy to participate in gamification interventions [30]. Some users may not respond positively to Gamification in certain contexts or with certain activities [22]. For example, according to [9], participants rated leaderboards least favorably in social networking contexts. Ethical issues related to Gamification in health and fitness tracking include privacy concerns, data security, and user autonomy [17]. The relationship between an intervention’s behavior change technique content and the resulting health behavior change is not simple and requires more study [37]. The fully mediating role of I.T. identity is necessary for gamified designs to encourage meaningful interactions with the apps [38]. There are some challenges in designing effective gamification strategies that appeal to both male and female users [39]. Physical limitations may hinder participation in gamification interventions [36]. Aesthetics in Gamification needs improvement, with realistic graphic visual and sound effects that provide more fun to users by creating aesthetically pleasing environments with sensory stimulation that are not at the required levels [40]. There is a challenge in implementing Gamification in physical activity applications [41] Gamified fitness apps may not be suitable for older adults due to usability concerns such as font size, color contrast, and navigation through the apps [42].

## Data Availability

Data is contained within the article.
